# The association of dietary inflammatory potential with sarcopenia in Chinese community-dwelling older adults

**DOI:** 10.1186/s12877-023-03938-7

**Published:** 2023-05-10

**Authors:** Dongsheng Bian, Chengjie Xuan, Xiyang Li, Wendi Zhou, Yaxiong Lu, Tianze Ding, Junhao Shen, Yongmei Shi, Guohong Li

**Affiliations:** 1 Department of Clinical Nutrition, Ruijin Hospital, Shanghai Jiao Tong University School of Medicine, Shanghai, 200025 China; 2 School of Public Health, Shanghai Jiao Tong University School of Medicine, Shanghai, 200025 China; 3 Department of Nutrition, College of Health Science and Technology, Shanghai Jiao Tong University School of Medicine, Shanghai, 200025 China

**Keywords:** Dietary inflammatory index, Sarcopenia, Muscle mass, Muscle performance, Community-Dwelling, Older adults

## Abstract

**Background:**

Dietary-induced inflammation is potentially associated with sarcopenia. Nevertheless, few studies have investigated the structure of the inflammatory diet and its correlation with muscle function and performance in both the upper and lower limbs. This study was performed to explore the association of the dietary inflammatory index (DII) with sarcopenia and its diagnostic parameters.

**Methods:**

We conducted a cross-sectional survey on a sample of 515 Chinese community-dwelling older adults selected through multistage cluster sampling from three districts in Shanghai. DII scores were calculated using a validated food frequency questionnaire. Sarcopenia and its diagnostic parameters were determined based on the definition set by the Asian Working Group on Sarcopenia (AWGS).

**Results:**

The mean age of study participants was 71.31 ± 4.71 years. The prevalence of sarcopenia in the cohort was 12.4%. Older adults in the highest DII quartile had a 3.339 times increased risk of sarcopenia compared to those in the lowest quartile (OR _Quartile4vs1_:3.339, 95%CI: 1.232, 9.052, *p*-trend: 0.004) after adjusting for confounding factors. Additionally, a more pro-inflammatory diet was associated with lower appendicular skeletal muscle index (ASMI) (OR _Quartile4vs1_: 3.005, 95%CI: 1.275, 7.318, *p*-trend: 0.005), a higher 5-times sit-stand test time score (OR _Quartile4vs1_: 4.942, 95%CI: 1.745, 13.993, *p*-trend: 0.005), and lower gait speed (OR _Quartile4vs1_: 2.392, 95%CI: 1.104, 5.185, *p*-trend: 0.041) after adjusting for confounding factors. However, there was no significant association between DII, handgrip strength, and Short Physical Performance Battery (SPPB) score in either the unadjusted or adjusted model.

**Conclusion:**

This study found that the association between consuming a more pro-inflammatory diet and sarcopenia in Chinese community-dwelling older adults was mainly due to underlying low intakes of dietary energy, protein, and anti-inflammatory foods, and not due to the high intake of pro-inflammatory foods. Meanwhile, DII was more highly correlated with lower limb muscle strength and performance compared to upper limb muscle strength.

**Supplementary Information:**

The online version contains supplementary material available at 10.1186/s12877-023-03938-7.

## Introduction

The structure of the world population is shifting towards a more aged population, with the proportion of the elderly increasing in almost all countries. In 2020, the number of the population aged over 65 already exceeded 700 million worldwide, with the number predicted to exceed 1.5 billion by 2050 [1]. This shift in the population structure disproportionately affects developing countries and regions. For example, by 2050, China is expected to experience a great increase in the elderly population, such that there will be 400 million Chinese citizens aged 65 + , 150 million of whom will be 80 + [2]. A consequence of this substantial demographic change will be a surge in the prevalence and incidence of age-associated diseases. Because sarcopenia is directly related to the aging process, the condition is becoming an issue of increasing concern, particularly with the global prevalence of sarcopenia ranging between 10 and 27% [3, 4]. Sarcopenia refers to the age-related progressive decline of muscle mass accompanied by impaired muscle strength and/or muscle function. The disorder is associated with higher rates of falls, decreased function, high mortality rates, and increased probability of hospitalization [3]. For any given individual, the prevalence of sarcopenia is expected to increase as the aging process accelerates over the lifespan. If the necessary measures are not taken to assist in the prevention and treatment of sarcopenia, it will inevitably become a huge burden on the healthcare system and social economy [5].

Inflammatory cytokines have been shown to prompt muscle wasting by stimulating protein catabolism and suppressing muscle synthesis. Elevated levels of pro-inflammatory cytokines in elderly individuals have been associated with an increased risk of sarcopenia. These include tumor necrosis factor-α (TNF-α), C-reactive protein (CRP), and interleukin (IL)-6 [6-8]. Dietary components may affect inflammation directly, for example, by counteracting the chronic low-grade inflammation associated with aging [9]. The Mediterranean diet consists of a dietary pattern involving a relatively high intake of whole grains, vegetables, fruits, nuts, and fish, which is associated with lower levels of inflammatory mediators (such as CRP and TNF-α) [9, 10]. In contrast, the Western-type dietary pattern is rich in refined sugars, salt, white flour, processed meats, and purified animal fats, which are associated with higher concentrations of inflammatory mediators [11].

The dietary inflammatory index (DII®) is a validated tool used to determine the inflammatory potential of an individual’s diet, which was developed based on a literature review exploring associations between dietary components and inflammatory mediators [12]. Higher and lower DII scores represent pro-inflammatory and anti-inflammatory diets, respectively. Many studies have been conducted to confirm that a pro-inflammatory diet may increase the risk of non-communicable diseases, such as obesity, diabetes, cancer, cardiovascular disease, and adverse mental health outcomes [13, 14]. Additionally, several studies have found that pro-inflammatory diets are associated with a decline in muscle mass and function [15-17]. However, different diagnostic criteria are used to identify sarcopenia across populations, countries, and regions, while different dietary patterns and cultural backgrounds also affect its prevalence. Yet, most of the relevant studies on this topic have been conducted in Middle Eastern or Western populations, while there have been few studies conducted in Asian populations, particularly in the elderly. As China transforms into an increasingly aged society, increasing significance lies in determining whether there exists an association between dietary inflammatory potential and sarcopenia in Chinese community-dwelling older adults. Hence, this cross-sectional study was conducted with the aim to explore the association of the DII score with sarcopenia and its diagnostic parameters. Furthermore, we analyze the dietary quality of Chinese community-dwelling older adults and further propose appropriate suggestions for the prevention of sarcopenia in this population.

## Methods

### Participants

This cross-sectional study was conducted from March 2022 to November 2022. Three districts were selected based on the geographical location and economic development of the 16 districts that comprise Shanghai. One town was then randomly selected from each of the three districts, and finally, 2—3 communities were selected from each town, namely Yang Jing town, Ban Song Yuan town, Chang Zheng town. The inclusion criteria were as follows: (1) aged 65 years and above; (2) ability to complete relevant tests and assessments independently; (3) signed an informed consent. But were excluded if they met any of the following criteria: (1) affected by loss of speech; (2) severely impaired in daily living activities; (3) the presence of metal implants (such as cardiac pacemakers). To ensure that the sample findings were valid for estimating the prevalence of sarcopenia in the Chinese community-dwelling older adults. The minimum sample size was calculated to be 451 older adults, assuming a prevalence of sarcopenia of 12%, at a 3% error rate [18] and 95% confidence interval. A total of 799 older adults were recruited for this cross-sectional study. However, the 236 older adults with uncompleted dietary assessment, 7 older adults who cannot complete body composition analysis and 41 older adults with missing anthropometric variables, hence 286 older adults were excluded. Finally, this study analysis comprised 515 community-dwelling old adults. This study was approved by the Human Ethics Research Committee of School of Public Health, Shanghai Jiao Tong University School of Medicine. Written informed consent was obtained from all study participants (No. SJUPN202008). Each participant signed an informed consent before examination.

### Dietary Inflammatory Index (DII)

A validated semiquantitative food frequency questionnaire (FFQ) comprised of 70 food-related items was used to assess the dietary intakes of participants through face-to-face interviews. All food-related items were divided into 14 food groups according to the nutritional characteristics of a given food. Three aspects of each item were listed in the FFQ, including whether the item was consumed, the usual frequency of consumption (number of times per day/week/month/year), and the estimated amount of food eaten each time, which was expressed using either the local unit ‘liang’ for weight (1 liang = 50 g) or the standard cup for volume (1 cup = 250 mL). All the researchers involved in conducting the food survey were dietitians who had worked in public hospitals for more than 5 years and were trained before conducting the study. Food models and pictures were also utilized to improve the accuracy of food recall by the participants. We estimated the average daily intakes of oil, salt, and sugar for the participants using the total household consumption of these condiments. Finally, we represented all food data as the daily intake per person and calculated individual nutrient intakes, including those of energy, protein, and carbohydrates, in accordance with the China Food Composition (National Institute of Nutrition and Food Safety, China CDC).

The DII was developed by Shivappa et al. [12, 13] based on a review of research literature conducted on participants from 11 countries across 4 continents, which explored the effects of 45 different food parameters on 6 inflammatory markers (IL-1 β, IL-4, IL-6, IL-10, TNF-α, and CRP). In this study, 28 out of the 45 food parameters were used to calculate DII. The 28 food parameters were carbohydrates, cholesterol, energy, total fat, fiber, folic acid, iron, magnesium, monounsaturated fatty acids, niacin, n-3 fatty acids, n-6 fatty acids, protein, polyunsaturated fatty acids, riboflavin, saturated fat, selenium, thiamin, vitamin A, vitamin C, vitamin D, vitamin E, zinc, isoflavones, alcohol, vitamin B12, vitamin B6, and β-carotene. The detailed DII calculation method has been described elsewhere [12, 19].

### Assessment of sarcopenia, muscle mass, and muscle function

Sarcopenia was diagnosed by considering the combination of low muscle mass and low muscle function (either strength or performance) according to the Asia Working Group on Sarcopenia (AWGS) 2019 consensus [20]. We used Inbody 770 (Biopace Co., Ltd., Seoul, Korea), a multifrequency bioelectrical impedance method, to conduct body composition analysis (such as body weight, basal metabolic rate, muscle mass, and fat mass), which has been validated in numerous Chinese populations. In this study, low muscle mass was diagnosed as an appendicular skeletal muscle index (ASMI) score of < 7.0 kg/m^2^ for men and < 5.7 kg/m^2^ for women via BIA. ASMI was calculated as appendicular skeletal muscle (ASM)/height^2^ (kg/m^2^).

Low muscle strength was diagnosed as handgrip strength of < 28 kg for men and < 18 kg for women. The handgrip strength measurement method has been described in detail in our previous study [19]. The results were measured 3 times on the dominant side (either the left or right hand) at 30-s intervals, with the maximum value considered the result. The results were expressed in kilograms of force (kg).

According to the AWGS (2019) [20], the criteria for low muscle performance is measured by any of the following tests: a 6 m walking speed (gait speed) of < 1.0 m/s, a 5-times sit-stand chair test (a surrogate for gait speed) time score of ≥ 12 s, or a Short Physical Performance Battery (SPPB) score of ≤ 9. In this study, low muscle performance was diagnosed by the latter test; an SPPB score of ≤ 9. The SPPB consists of 3 physical performance tests used to assess the frailty domains of balance (static standing balance), gait speed, and weakness (5-times sit-stand chair test). For the balance tests, participants were instructed to maintain their feet in side-by-side, semi-tandem, and tandem positions for 10 s each. For the gait speed assessment test, each participant’s usual speed was timed during a 4 m walk. For the 5-times sit-stand chair test, participants were instructed to stand up and sit down 5 times as quickly as possible.

### Covariates

We developed a survey questionnaire for this study, which was then used to obtain the socio-demographic characteristic data of the participants (age, sex, educational background, annual household income, urban or rural, etc.).

#### Cognitive impairment

We assessed cognitive impairment in the community-dwelling older adults using a pre-developed and validated computerized neuropsychological assessment device—"Memory Guard", which was described in detail in the previous study [21]. The Memory Guard included 38 features (17 features of option score, 20 features of time score, and 1 demographic feature). It uses computerized tests designed with immersive content. Besides this feature engineering are incorporated into the model to further improve classification accuracy. The Memory Guard was presented in the form of an app, and operated through mobile phones or tablets with a touch-screen using the Android or IOS system. The completion of the entire test system was expected to be approximately 20 min. Computerized tool automatically identifies whether older adults have a risk of cognitive impairment.

#### Fall risk

The Morse Fall Scale was used to assess fall risk. This scale consists of 6 items reflecting risk factors of falling, including history of falling, secondary diagnosis, ambulatory aids, intravenous therapy, type of gait, and mental status. The total score ranges between 0 and 125, with a cut-off score of ≥ 45. In this study, we defined ≥ 45 as indicative of a high fall risk, 25—45 as indicative of a moderate fall risk, and < 25 as indicative of a low fall risk [22].

#### Social support

Social support was assessed using the Social Support Rate Scale (SSRS), which was designed to determine how much support respondents receive from their family, friends, and various social contexts. SSRS consists of 3 subscales: subjective support (including the number of friends who have offered assistance, relationships with neighbors, relationships with colleagues, and the level of support from family members), objective support (problem-solving channels in emergencies and sources of psychological comfort in circumstances where participants may face pressure or resistance), and support utilization (the way in which a participant expresses themselves when in trouble, the way in which they seek help when in trouble, and the degree of their willing participation in group activities). The total SSRS score is the sum of these 3 subscale scores, with a higher score indicating a higher level of social support [23]. The total support score is classified into 3 categories: low (≤ 22), moderate (23—44), and high (≥ 45) levels of support [24].

#### Geriatric depression

Geriatric depression was measured using a Chinese version of the Geriatric Depression Scale (GDS-15). The total GDS-15 score is calculated as the sum of the 15 items, with higher scores corresponding to greater levels of depression. Depressive disorders were defined using the cutoff points for community-dwelling elders: < 5, normal; 5—9, minor depressive disorder; ≥ 10, moderate and severe depressive disorder [25].

### Statistical analysis

SPSS22.0 was used for all statistical analyses. We grouped the DII into quartiles and assessed the baseline characteristics of the participants. Continuous variables were shown as the means and standard errors. Categorical variables were shown as percentages. The variables were analyzed using ANOVA and Chi-square tests for continuous and categorical variables, respectively. Binary logistic regression was used to determine the association between DII and sarcopenia in crude and adjusted models. Model 1 was an unadjusted regression model. Model 2 was adjusted for age (continuous), BMI (continuous), gender (male and female), and drinking history (yes and no). Some studies have shown a correlation between cognitive impairment [26, 27], depression [28, 29] and fall risk [30] with sarcopenia, so we further included these factors as adjustment variables in Model 3. Some studies have also shown a correlation between social support and diet quality and muscle strength trajectories in older adults [31, 32]. Furthermore, studies have shown that some specific diseases increase the risk of sarcopenia, such as type 2 diabetes mellitus (T2DM) [33], hypertension [34], cardiovascular disease (CVD) [35], cancer [36]. Therefore, we included social support as an adjustment variable. Further adjustments were made for education level (high school and below, college degree, bachelor’s degree and above, and master’s degree and above), T2DM (yes and no), hypertension (yes and no), CVD (yes and no), cancer (yes and no), cognitive impairment (yes or no), level of depression (none, minor, moderate, and severe), fall risk (low, moderate, and high), levels of social support (low, moderate, and high), and total energy intake (continuous) in Model 3. Additionally, we conducted a sensitivity analysis to further validate the correlation between DII and sarcopenia, using European Working Group on Sarcopenia 2 (EWGSOP2) for the diagnostic criteria of sarcopenia (Supplemental Table 1S and Table 2S) [37]. Trends of associations were assessed using the DII quartiles as continuous variables.

## Results

### Characteristics of the participants

The general characteristics of study participants with and without sarcopenia are shown in Table 1. A total of 203 males and 312 females were included in this study. Their mean age was 71.31 ± 4.71 years. Of them, 64 (12.4%) had sarcopenia, 58 had a low SPPB score, 92 had lower muscle performance (gait speed < 1.0 m/s), and 57 had low muscle performance (≥ 12 s on the 5-times sit-stand chair test). 33.8% older adults had cognitive impairment. Participants who had sarcopenia were relatively older and had lower BMI, ASMI, FFMI, FMI, handgrip strength, SPPB score, and muscle performance values when compared to those without sarcopenia.

**Table 1:** Characteristics of individuals with and without sarcopenia

	All	No Sarcopenia (*n* = 451)	Sarcopenia (*n* = 64)	*p*-value
Age	71.31 ± 4.71	71.08 ± 4.62	73.21 ± 5.03	0.002^*^
Gender				
Male	203	181	22	0.229
Female	312	270	42	
Education level				
High school and below	34	28	6	0.125
College degree	336	300	36	
Bachelor’s degree and above	92	86	6	
Master’s degree and above	53	44	9	
Hight (cm)	162.26 ± 7.75	162.76 ± 7.68	158.24 ± 7.14	0.000^*^
Weight (kg)	62.31 ± 10.74	63.44 ± 10.46	53.22 ± 8.46	0.000^*^
BMI (kg/m^2^)	23.59 ± 3.21	23.89 ± 3.17	21.16 ± 2.52	0.000^*^
Drinking history (%)				0.216
Yes	67	62	5	
No	448	396	52	
Annual household income (RMB)				0.215
≤ 30,000	44	41	3	
30,000—90,000	394	348	46	
90,000—240,000	71	65	6	
> 240,000	6	4	2	
Lives alone				0.193
Yes	21	17	4	
No	494	441	53	
FFMI (kg/m^2^)	16.60 ± 1.85	16.80 ± 1.811	15.03 ± 1.38	0.000^*^
BMR (kcal)	1323.12 ± 171.19	1339.80 ± 168.45	1189.09 ± 129.87	0.000^*^
FMI (kg/m^2^)	6.96 ± 2.46	7.06 ± 2.49	6.14 ± 2.09	0.000^*^
ASMI (kg/m^2^)	6.86 ± 1.02	6.98 ± 0.99	5.84 ± 0.70	0.000^*^
Handgrip strength (kg)	26.08 ± 8.02	26.92 ± 7.93	19.30 ± 4.85	0.000^*^
Morse score	13.73 ± 15.43	13.62 ± 15.19	14.56 ± 17.33	0.698
SSRS score	35.05 ± 7.54	35.15 ± 7.55	34.26 ± 7.54	0.404
GDS-15 score	1.72 ± 2.04	1.69 ± 1.94	1.98 ± 2.68	0.278
SPPB score	11.11 ± 1.49	11.22 ± 1.37	10.25 ± 2.06	0.000^*^
Cognitive impairment				0.028^*^
Yes	174	145	29	
No	341	306	35	
T2DM				0.269
Yes	91	82	9	
No	424	369	55	
Hypertension				0.534
Yes	232	203	29	
No	283	248	35	
CVD				0.255
Yes	66	60	6	
No	449	391	58	
Cancer				0.594
Yes	11	10	1	
No	504	441	63	
Low SPPB				0.000^*^
Yes	58	41	17	
No	457	417	40	
Low 6 m walk test (gait speed)				0.002^*^
Yes	92	73	17	
No	423	385	38	
Low 5-times sit-stand chair test				0.000^*^
Yes	57	41	16	
No	458	417	41	
DII score	0.20 ± 1.75	0.10 ± 1.70	0.94 ± 1.93	0.001^*^

Data are presented as mean ± SD for continuous variables and n for categorical variables. ANOVA test for continuous variables and Chi-square test for categorical variables

*Abbreviations*: *BMI* Body mass index, *FFMI* Fat free mass index, *FMI* Fat mass index, *BMR* Basal metabolic rate, *FMI* Fat mass index, *ASMI* Appendicular skeletal muscle index, *GDS* Geriatric Depression Scale, *SSRS* Social Support Rate Scale, *SPPB* Short Physical Performance Battery, *T2DM* type 2 diabetes mellitus, *CVD* Cardiovascular disease, *DII* Dietary inflammatory index. Low SPPB defined as SPPB score ≤ 9. Low gait speed defined as < 1.0 m/s. Low 5-times sit-stand chair test defined as ≥ 12 s, *DII* Dietary inflammatory index

^*^p < 0.05

The mean ± SD and range of the DII scores in the study participants (*n* = 515) were 0.19 ± 1.75 and − 4.58 to 4.75, respectively. The clinical and demographic characteristics of patients according to the DII quartiles are presented in Table 2. Lower handgrip strength, fat-free mass, and 5-times sit-stand chair test time scores were observed among the older adults in the highest quartile of the dietary inflammatory index score, which characterizes them as consuming a more pro-inflammatory diet. Although there was a trend towards lower ASMI and SPPB scores with increasing DII score, this correlation did not attain statistical significance. Furthermore, no differences in age, gender, comorbidities, GDS-15 score, or annual household income were observed between the different groups.

**Table 2:** Participant characteristics according to DII quartiles

Variable	Quartile 1 (*n* = 128)	Quartile 2 (*n* = 129)	Quartile 3 (*n* = 129)	Quartile 4 (*n* = 129)	*p*
Age (years)	71.32 ± 4.47	71.55 ± 4.45	71.43 ± 5.12	70.96 ± 4.79	0.785
Gender					0.288
Male	59	48	45	51	
Female	69	81	84	78	
Education level					0.013^*^
High school and below	4	4	13	13	
College degree	88	78	78	92	
Bachelor’s degree and above	20	28	25	19	
Master’s degree and above	16	19	13	5	
BMI (kg/m^2^)	23.65 ± 2.91	23.19 ± 3.31	23.67 ± 3.26	23.79 ± 3.32	0.446
Comorbidities					0.072
No	54	53	49	36	
Yes	74	76	80	93	
Drinker (%)					0.039^*^
Yes	26	12	15	15	
No	102	117	114	114	
Annual household income (RMB)					0.298
≤ 30,000 RMB	9	12	9	14	
30,000—90,000	99	92	104	99	
90,000—240,000	18	22	15	16	
> 240,000	2	3	1	0	
Lives alone					
Yes	10	7	12	12	0.622
No	118	122	117	117	
FFM (kg)	46.25 ± 8.10	43.26 ± 8.10	43.62 ± 7.82	43.17 ± 7.77	0.005^*^
FFMI (kg/m^2^)	16.94 ± 1.86	16.40 ± 1.85	16.58 ± 1.78	16.61 ± 1.73	0.122
BMR (kcal)	1366.68 ± 172.91	1305.88 ± 169.04	1313.64 ± 169.04	1307.16 ± 168.28	0.011^*^
FM (kg)	17.92 ± 5.72	17.44 ± 6.82	18.65 ± 6.24	18.70 ± 5.68	0.288
FMI (kg/m^2^)	6.69 ± 2.19	6.65 ± 2.67	7.18 ± 2.60	7.13 ± 2.20	0.162
ASMI (kg/m^2^)	7.07 ± 1.01	6.77 ± 1.11	6.84 ± 0.99	6.75 ± 0.95	0.064
Handgrip strength (kg)	27.56 ± 8.64	26.10 ± 8.56	25.59 ± 7.73	24.84 ± 6.99	0.048^*^
Morse score	13.40 ± 16.05	12.83 ± 14.35	14.03 ± 15.21	14.65 ± 16.17	0.800
SSRS score	35.39 ± 7.38	36.29 ± 7.47	34.66 ± 7.31	33.88 ± 7.87	0.067
GDS-15 score	1.51 ± 2.13	1.67 ± 1.72	1.66 ± 1.94	2.05 ± 2.33	0.172
SPPB score	11.23 ± 1.36	11.16 ± 1.45	11.09 ± 1.38	10.98 ± 1.74	0.571
T2DM					0.470
Yes	21	25	18	27	
No	107	103	111	103	
Hypertension					0.040
Yes	49	52	60	71	
No	79	76	69	59	
CVD					0.548
Yes	14	14	17	21	
No	114	114	112	109	
Cancer					0.627
Yes	1	3	4	3	
No	127	125	125	127	
Cognitive impairment					0.879
Yes	41	42	47	44	
No	87	87	82	85	
Low SPPB					0.474
Yes	11	14	19	14	
No	117	115	110	115	
Low 6 m walk test (gait speed)					0.147
Yes	15	25	23	23	
No	113	104	106	100	
Low 5-times sit-stand chair test					0.023^*^
Yes	6	17	13	21	
No	122	112	116	108	

Data are presented as mean ± SD for continuous variables and n for categorical variables. ANOVA test for continuous variables and Chi-square test for categorical variables

*Abbreviations*: *DII* Dietary inflammatory index, *BMI* Bbody mass index, *FFMI* Fat free mass index, *FMI* Fat mass index, *BMR* Basal metabolic rate, *FMI* Fat mass index, *ASMI* Appendicular skeletal muscle index, *GDS* Geriatric Depression Scale, *SSRS* Social Support Rate Scale, *SPPB* Short Physical Performance Battery, *T2DM* type 2 diabetes mellitus, *CVD* Cardiovascular disease. Low SPPB defined as SPPB score ≤ 9. Low gait speed defined as < 1.0 m/s. Low 5-times sit-stand chair test defined as ≥ 12 s

^*^p < 0.05

### Nutrient and food intake according to DII quartiles

Table 3 shows the distribution of nutrient and food composition across quartiles of the DII score among community-dwelling older adults. Participants in the fourth quartile were characterized by lower intakes of anti-inflammatory foods and nutrients, such as protein, PUFA, MUFA, n-3 fatty acids, isoflavones, vitamins, and minerals, when compared to those in the first quartile. Similarly, participants who consumed a pro-inflammatory diet (those in the highest DII quartile) had lower total energy, carbohydrate, saturated fat, and fiber intakes as compared to those consuming an anti-inflammatory diet (those in the lowest DII quartile).

**Table 3:** Nutrient and food intakes according to DII quartiles

Variable	Quartile 1 (*n* = 128)	Quartile 2 (*n* = 129)	Quartile 3 (*n* = 129)	Quartile 4 (*n* = 129)	*p*
Total energy (kcal)	1595.27 ± 375.33	1415.01 ± 385.49	1288.85 ± 409.94	1177.77 ± 334.84	0.000^*^
Protein (g)	77.90 ± 20.32	59.06 ± 12.20	50.22 ± 12.37	42.81 ± 10.79	0.000^*^
Carbohydrate (g)	184.37 ± 52.69	169.02 ± 51.32	152.07 ± 55.09	142.16 ± 48.78	0.000^*^
Total Fat (g)	66.36 ± 21.20	62.07 ± 26.64	59.37 ± 26.20	53.36 ± 20.31	0.000^*^
Saturated fat (g)	12.08 ± 6.62	10.35 ± 6.28	9.12 ± 3.91	7.04 ± 3.91	0.000^*^
Fiber (g)	20.75 ± 5.71	15.41 ± 3.26	12.97 ± 3.00	9.53 ± 3.59	0.000^*^
PUFA (g)	6.04 ± 2.83	4.21 ± 1.90	3.89 ± 2.14	2.70 ± 1.81	0.000^*^
MUFA (g)	9.20 ± 3.60	7.00 ± 2.18	6.21 ± 2.08	5.20 ± 1.95	0.000^*^
n-3 Fatty acids (g)	0.61 ± 0.32	0.40 ± 0.17	0.33 ± 0.15	0.22 ± 0.10	0.000^*^
n-6 Fatty acids (g)	6.96 ± 4.60	5.08 ± 2.38	4.14 ± 1.92	2.90 ± 1.87	0.000^*^
Vitamin B1 (mg)	0.97 ± 0.32	0.70 ± 0.18	0.59 ± 0.28	0.54 ± 0.35	0.000^*^
Vitamin B2 (mg)	1.78 ± 0.61	1.38 ± 0.44	1.18 ± 0.45	0.87 ± 0.44	0.000^*^
Vitamin B6 (mg)	41.73 ± 104.48	25.29 ± 35.94	21.89 ± 37.17	12.62 ± 26.81	0.001^*^
Vitamin B12 (µ g)	5.17 ± 12.46	2.47 ± 1.04	2.39 ± 2.42	3.12 ± 14.13	0.069
Vitamin C (mg)	139.19 ± 47.62	99.85 ± 23.22	73.51 ± 20.24	57.55 ± 22.29	0.000^*^
β-Carotene ( µ g)	13,387.71 ± 4841.33	9634.17 ± 3593.36	6660.67 ± 2873.82	4570.68 ± 2740.96	0.000^*^
Vitamin D (µ g)	5.45 ± 2.11	4.13 ± 1.48	3.73 ± 1.73	2.79 ± 1.46	0.000^*^
Vitamin E (mg)	17.68 ± 4.88	12.90 ± 2.45	10.55 ± 1.70	7.59 ± 1.99	0.000^*^
Niacin (mg)	17.68 ± 5.12	14.08 ± 3.94	11.95 ± 4.10	10.94 ± 3.73	0.000^*^
Folic acid (µ g)	433.30 ± 115.07	310.81 ± 70.17	244.68 ± 51.15	190.23 ± 47.64	0.000^*^
Isoflavones (mg)	11.01 ± 10.32	7.29 ± 5.83	5.13 ± 4.71	2.74 ± 3.19	0.000^*^
Mg (mg)	397.55 ± 85.57	295.41 ± 44.37	244.09 ± 45.37	190.82 ± 43.49	0.000^*^
Se (mg)	47.03 ± 17.16	34.64 ± 9.01	31.36 ± 9.80	26.27 ± 8.96	0.000^*^
Zn (mg)	13.25 ± 3.86	10.53 ± 2.61	8.85 ± 2.04	7.70 ± 2.19	0.000^*^
Grains and grain products (g)	294.59 ± 155.27	309.83 ± 163.17	272.10 ± 170.40	288.37 ± 171.31	0.329
Soybean and its products (g)	103.71 ± 114.09	73.52 ± 71.36	54.50 ± 63.96	29.92 ± 46.22	0.000^*^
Vegetables (g)	537.54 ± 191.47	386.85 ± 99.09	279.42 ± 75.31	226.67 ± 84.50	0.000^*^
Fruits (g)	202.31 ± 172.05	180.42 ± 96.44	191.71 ± 162.36	148.47 ± 139.35	0.020^*^
Dairy products (g)	266.44 ± 138.87	204.05 ± 129.21	185.69 ± 123.74	131.24 ± 111.98	0.000^*^
Eggs (g)	50.60 ± 18.07	45.91 ± 14.88	47.15 ± 17.74	47.21 ± 17.06	0.056
Poultry and Meat (g)	82.98 ± 50.91	62.40 ± 39.71	51.58 ± 36.49	61.71 ± 42.47	0.000^*^
Fish and shrimp (g)	80.34 ± 72.67	51.85 ± 35.55	46.30 ± 37.70	38.33 ± 33.71	0.000^*^
Snack (g)	31.53 ± 26.62	25.05 ± 30.78	25.52 ± 31.46	16.53 ± 20.90	0.000^*^
Alcohol (g)	14.53 ± 50.13	9.73 ± 45.81	10.36 ± 50.63	18.84 ± 95.45	0.638
Edible oil (g)	27.12 ± 14.11	28.39 ± 14.50	29.70 ± 15.66	31.10 ± 16.41	0.181
Salt (g)	4.80 ± 3.92	4.41 ± 2.61	4.84 ± 2.64	4.88 ± 3.01	0.599
Water (ml)	1203.13 ± 776.81	1188.62 ± 691.94	1061.24 ± 691.02	1051.56 ± 606.13	0.156
Sugar (g)	2.44 ± 3.11	2.74 ± 3.25	3.02 ± 3.26	3.44 ± 4.34	0.133

Data are presented as mean ± SD for continuous variables. ANOVA test for continuous variables

*Abbreviations*: *MUFA* Monounsaturated fatty acid, *PUFA* Polyunsaturated fatty acid

^*^p < 0.05

### Association between DII and sarcopenia among community-dwelling older adults

Table 4 presents the associations between the DII score and sarcopenia, as well as its diagnostic parameters (muscle mass and performance). In the unadjusted model, the risk of sarcopenia increased by 2.187 times in older adults consuming a pro-inflammatory diet (OR _Quartile4vs1_:2.187, 95%CI: 1.013, 4.722, *p*-trend: 0.015). After adjusting for confounding factors, older adults in the highest DII quartile had a 3.339 times increased risk of sarcopenia when compared to those in the lowest quartile (OR _Quartile4vs1_:3.339, 95%CI: 1.232, 9.052, *p*-trend: 0.004). Moreover, a more pro-inflammatory diet was associated with a lower ASMI score in both the unadjusted and adjusted models (OR _Quartile4vs1_: 1.972, 95%CI: 1.053, 3.694 *p*-trend: 0.023, model 1; OR _Quartile4vs1_: 3.055, 95%CI: 1.275, 7.318, *p*-trend: 0.005, model 3; respectively). An association was also observed between the highest DII quartile and higher sit-stand chair test time scores in both the unadjusted and adjusted models (OR _Quartile4vs1_: 3.954, 95%CI: 1.539, 10.157, *p*-trend: 0.010, model 1; OR _Quartile4vs1_: 4.942, 95%CI: 1.745, 13.993, *p*-trend: 0.005, model 3; respectively). A positive association was observed between the highest DII quartile and lower gait speed in both the unadjusted and fully adjusted models (OR _Quartile4vs1_: 2.185, 95%CI: 1.108, 4.308, *p*-trend: 0.040, model 1; OR _Quartile4vs1_: 2.392, 95%CI: 1.104, 5.185, *p*-trend: 0.042, model 3; respectively). However, there was no significant association found between DII score, muscle strength (handgrip strength), and muscle performance (SPPB) in either the unadjusted or adjusted models.

**Table 4:** Multivariable-adjusted odds ratios (95% CIs) for sarcopenia and its diagnostic parameters across DII quartile categories

Model	DII, OR (95%CI)				***P*-trend**
	Quartile 1 (*n* = 128)	Quartile 2 (*n* = 129)	Quartile 3 (*n* = 129)	Quartile 4 (*n* = 129)	
Sarcopenia					
Model 1	1	0.992 (0.414, 2.376)	1.952 (0.894, 4.260)	2.187 (1.013, 4.722)^*^	0.015
Model 2	1	0.789 (0.302, 2.065)	2.089 (0.883, 4.943)	2.618 (1.111, 6.169)^*^	0.003
Model 3	1	0.829 (0.301, 2.281)	2.582 (1.000, 6.669)^*^	3.339 (1.232, 9.052)^*^	0.004
Low muscle mass (ASMI)					
Model 1	1	1.245 (0.641, 2.418)	1.590 (0.837, 3.023)	1.972 (1.053, 3.694)^*^	0.023
Model 2	1	1.163 (0.530, 2.550)	2.005 (0.931, 4.321)	2.961 (1.384, 6.334)^*^	0.002
Model 3	1	1.181 (0.522, 2.675)	2.131(0.923, 4.917)	3.055 (1.275, 7.318)^*^	0.005
Low muscle strength (handgrip strength)					
Model 1	1	0.990 (0.555, 1.764)	1.033 (0.582, 1.836)	1.169 (0.664, 2.060)	0.572
Model 2	1	0.963 (0.530, 1.751)	1.066 (0.589, 1.928)	1.248 (0.696, 2.240)	0.413
Model 3	1	0.965 (0.522, 1.782)	1.070 (0.571, 2.006)	1.210 (0.629, 2.329)	0.529
Low muscle performance (SPPB)					
Model 1	1	1.295 (0.564, 2.971)	1.837 (0.836, 4.035)	1.295 (0.564, 2.971)	0.410
Model 2	1	1.380 (0.587, 3.246)	1.870 (0.829, 4.216)	1.394 (0.593, 3.276)	0.120
Model 3	1	1.332 (0.559, 3.175)	1.831 (0.783, 4.278)	1.355 (0.538, 3.413)	0.352
Low muscle performance (6 m walk test)					
Model 1	1	1.811 (0.905, 3.622)	1.635 (0.810, 3.300)	2.185 (1.108, 4.308)^*^	0.040
Model 2	1	1.759 (0.862, 3.588)	1.510 (0.730, 3.125)	2.154 (1.067, 4.350)^*^	0.044
Model 3	1	1.864 (0.895, 3.880)	1.718 (0.798, 3.696)	2.392 (1.104, 5.185)^*^	0.042
Low muscle performance (5-times sit-stand chair test)					
Model 1	1	3.086 (1.175, 8.104)^*^	2.279 (0.838, 6.195)	3.954 (1.539, 10.157)^*^	0.010
Model 2	1	2.935 (1.107, 7.778)^*^	2.143 (0.781, 5.881)	3.817 (1.474, 9.889)^*^	0.013
Model 3	1	3.321 (1.216, 9.068)^*^	2.644 (0.917, 7.628)	4.942 (1.745, 13.993)^*^	0.005

Odds ratios and 95% confidence intervals were estimated by logistic regression models with different levels of adjustment. Model 1: unadjusted. Model 2: adjusted for age, BMI, gender, drinking history, and living alone. Model 3: additionally adjusted for education level, T2DM, hypertension, CVD, cancer, cognitive impairment, level of depression, fall risk, SSRS, and total energy intake

*Abbreviations*: *DII* Dietary inflammatory index, *OR* Odds ratio, *CI* Confidence interval, *ASMI* Appendicular skeletal muscle index, *SPPB* Short Physical Performance Battery, *BMI* Body mass index, *T2DM* Type 2 diabetes mellitus, *CVD* Cardiovascular disease, *SSRS* Social Support Rate Scale

^*^p < 0.05

## Discussion

A previous meta-analysis reported that the pooled prevalence of sarcopenia was 12.9% in males and 11.2% in females in community-dwelling Chinese older adults aged 60 years and over [38]. Multistage sampling was conducted to select a sample of older people in the community in order to make the sample more representative. Our study showed similar results, with an overall prevalence rate of 12.4% in older adults with sarcopenia ages 65 years and over (male: 10.8%, female: 13.5%). Additionally, more potential adjustment variables have been included to ensure that the results are more credible. Our findings also reveal that a more pro-inflammatory diet is associated with an increased risk of sarcopenia in this cohort of older adults. This association remained after adjusting for potential confounding factors. After using the EWGSOP2 diagnosis criteria, we also found a correlation between DII and sarcopenia. Moreover, this is the first study investigating the association between a pro-inflammatory diet and sarcopenia in community-dwelling Chinese older adults. Some previous studies have reported on the associations of the DII score with sarcopenia, and our findings show similar results. An Iranian study enrolled 300 older adults and found that those with the highest DII scores were 2.18 times (95% CI: 1.01–4.74) more likely to have sarcopenia than those with the lowest DII scores [15]. A total of 6,082 participants from the National Health and Nutrition Examination Survey (NHANES) study also found that higher DII scores were associated with sarcopenia (OR: 1.17, 95%CI:1.00, 1.37) [16]. A sterile, chronic, and low-grade form of systemic inflammation known as “inflammaging” occurs during the process of aging in the elderly [39]. Much evidence has indicated that this aging-associated form of low-grade chronic inflammation may be a key factor in the pathophysiology of sarcopenia [40-42]. Earlier studies confirmed the correlation between DII scores and the degree of bodily inflammation [13, 43]. This further supports the possibility that a pro-inflammatory diet exacerbates the risk of sarcopenia.

Further analysis was then conducted to examine the correlation between DII scores and the main parameters of the diagnosis of sarcopenia. We found that a more pro-inflammatory diet correlated with lower muscle mass. However, available existing studies on the correlation between DII and ASMI scores in older adults have yielded inconsistent results. In a cross-sectional study conducted on 800 individuals aged 60—95 years in Australia, a more pro-inflammatory diet was associated with lower muscle mass [44]. Findings from a study of National Health and Nutrition Examination Survey data reported that the E-DII score was associated with a lower ASMI score in multivariable-adjusted models [16]. In contrast, a cross-sectional study conducted on 300 elderly people indicated that subjects in the top tertile for DII scores had no significantly greater odds of low muscle mass [15]. These inconsistencies may be due to a range of factors, including employing different means of measuring muscle mass, inconsistent diagnostic criteria for ASMI, an insufficient sample size, different age ranges in the sample, and different ranges of DII scores.

In our investigation, comprehensive measurements of muscle strength and muscle performance; including handgrip strength, the SPPB, the 6 m walk test, and the 5-times sit-stand chair test; were carried out in accordance with the AWGS definition of sarcopenia, signifying its first instance of validation in Chinese community-dwelling older adults. The results showed that a more pro-inflammatory diet was associated with lower levels of muscle performance (especially as determined by gait speed and the sit-stand chair test). However, no significant association was observed between the DII score, handgrip strength, and the SPPB score. Handgrip strength, SPPB, and gait speed, which are clinical markers of poor mobility, were used in this study to assess low muscle function for the diagnosis of sarcopenia. Several studies also have explored the relationship between the DII score and these markers, but results have been inconsistent. In a study conducted on 1,948 community-dwelling individuals aged ≥ 60 years from the Seniors-ENRICA cohort, the results showed that DII scores were associated with slow gait speed in the SPPB test but were not associated with SPPB test scores or sit-stand chair test scores [45]. Furthermore, Corey et al. studied 173 older adults aged 65—85 years and found that the DII score was negatively associated with handgrip strength and muscle mass after adjusting for covariates [46]. In a South Korean study of 321 participants aged 70—85 years old, slow gait speed and low handgrip strength were positively associated with higher DII scores. Similarly, no statistically significant correlation was found between the DII score and sit-stand chair test times [47]. Conversely, no significant associations have been observed between the DII score and either gait speed or handgrip strength in other studies [15, 48].

Systemic inflammation may serve as an early marker to identify individuals at risk for gait speed decline [45, 49]. In a longitudinal study conducted to explore the associations of a pro-inflammatory pattern of cytokines in the body (measured two decades prior) with gait speed among community-dwelling adults, it was found that gait speed was negatively associated with CRP levels after adjusting for baseline and concurrent cardiometabolic risk factors [49]. This finding can be explained by the interplay between two pathways [50, 51]: “sickness behavior” and “brain aging.” In addition, our results indicate that the degree of physical ability measured by the sit-stand chair test and gait speed test (which each assess lower limb strength and function) were negatively associated with consuming a more pro-inflammatory diet, but handgrip strength was not. The results of a South Korean population study are like those obtained by the present study [52], in that they suggest that physical ability measured by the sit-stand chair test correlates with abilities measured by the gait speed test and handgrip strength test, though it seems to be a better proxy of the former (gait speed) than the latter (handgrip strength). The sit-stand chair test is considered an effective means to measure lower body strength in community-residing older adults [53]. The AWGS 2019 guidelines recommend the sit-stand chair test be performed for evaluating physical performance. The EWGSOP2 [37] proposed that the sit-stand chair test is a qualified and convenient measure of muscle strength. Additionally, EWGSOP2 suggested that grip strength could be used as a reliable proxy for more complicated arm and leg strength measurements, while the sit-stand chair test could be used as a reliable proxy for assessing leg muscle strength (pertaining to the quadriceps muscle group) [53]. Thus, a pro-inflammatory diet may initially induce sarcopenia by exacerbating the age-associated decline in lower limb muscle function.

The average energy intake of community-dwelling Chinese older adults in this survey was 1,368.32 ± 406.99 kcal, which is lower than the recommended intake of the Chinese Dietary Guidelines. Moreover, the average energy intake was 1,595.27 ± 375.33 in participants consuming an anti-inflammatory diet (Quartile 1). Inadequate intakes of protein and anti-inflammatory nutrients is a possible factor involved in the development of sarcopenia [54-56]. We found that the intakes of protein, carbohydrates, n-3 PUFA, vitamins, minerals, and phytochemicals were lower in participants consuming a pro-inflammatory diet (Quartile 4) than in those consuming an anti-inflammatory diet group (Quartile 1). The average protein intake of the highest quartile was 0.71 ± 0.22 g/kg·d, which fails to meet the recommendations set for healthy older individuals. Our findings support those observed by Son et al., who suggested that the association between the DII score and sarcopenia was mainly due to a low intake of anti-inflammatory foods, and not due to the high intake of pro-inflammatory foods [57]. The dietary structure of the pro-inflammatory diet calculated by DII in the Western older adults was different from that of the Chinese older adults, pro-inflammatory diets in Western populations are more commonly caused by a high intake of pro-inflammatory foods [44, 58]. For example, Corley et al. found that the UK older adults with pro-inflammatory diet had higher intake of total energy, carbohydrate, total fat, saturated fat, and cholesterol, but lower intake of protein, PUFA and vitamins [59]. A study from the 2015 China Adult Chronic Disease and Nutrition Surveillance showed that among 18,161 older adults aged 65 and above, 75.8% had inadequate energy intake and 76.6% had inadequate protein intake [60]. This evidence suggests that the poor dietary intakes of Chinese older adults are an urgent issue to address, as they are likely to result in malnutrition and sarcopenia, which cause significant adverse effects on well-being.

Although sarcopenia represents a growing concern among researchers and clinicians around the world, the condition may be relatively less prioritized in healthcare in developing countries, particularly in China, which is characterized by a large and growing elderly population. Boshnjaku [5] considered that healthcare providers from lower- and middle-income countries are currently unprepared for a rise in the prevalence of sarcopenia and lack the resources to deal with it. Therefore, it is crucial to screen for specific influencing factors in the older population with sarcopenia. Firstly, in this study, we found that the pro-inflammatory diet in the elderly population is mainly due to inadequate intake, which will exacerbate the incidence of malnutrition; also, malnutrition is an independent risk factor for sarcopenia [61, 62]. Thus, it is essential for older adults with sarcopenia to be screened and assessed for malnutrition primarily, using several tools including the Mini Nutritional Assessment, Nutritional Risk Screening, Risk Evaluation for Eating and Nutrition, et al. Secondly, older adults with dysphagia may have a deterioration in muscle mass and strength of generalized skeletal muscles and swallowing-related muscles, the main manifestations are a reduction in the thickness of the tongue, chin hyoid muscle and pharyngeal wall, a reduction in tongue pressure and a weakened pharyngeal contraction [61]. Thus, it is also essential to screen for dysphagia in the older adults, such as using the 10-Item Eating Assessment Tool. Thirdly, poor oral health was also a determinant of sarcopenia. The interplay between oral diseases and sarcopenia may be explained through biological and environmental factors that are linked to the common burden of inflammation and oxidative stress [63]. So, it is also important for the older adults to focus on their oral problems, such as periodontal disease and edentulism, et al. As above, nutrition interventions are important determinants in the management of sarcopenia in older adults. Expert consensus on the management of sarcopenia in China was updated in 2021, which emphasized the role of protein supplement and nutritional screening in the prevention and treatment of sarcopenia, while not mentioning issues concerning dietary quality, such as energy intake. In addition, we need to pay attention to the quality of the diet of the elderly, especially the adequate intake of essential nutrients such as energy, protein, fluid, and micronutrients. The texture of food products should be adjusted for chewing and swallowing to avoid the risk of choking [63]. Oral nutritional supplements should be considered when the intake of older people is inadequate [56]. Healthcare providers could disseminate a healthy nutrition plan for community-dwelling older adults by employing a range of tools, including the use of health education and health promotion.

Several limitations of this work should be addressed. Firstly, this study used only 25 nutrients to calculate the DII score. Nevertheless, the correlation between DII scores and inflammatory parameters remained unchanged in other relevant studies that included only 23—30 food parameters [44, 57, 64]. Secondly, although we selected three communities for participation in our survey, the sample size was still small. Hence, our findings are not necessarily representative of the wider Chinese community of dwelling older adults. Thirdly, our study found that the DII score was more highly associated with muscle mass, lower limb muscle function, and lower limb muscle performance. However, our study is only a cross-sectional investigation, and it cannot indicate whether there is a causal relationship between DII and sarcopenia, so further prospective cohort studies are needed to validate our findings. Fourthly, we probably did not consider other potential confounding factors, such as physical activity, chronic pain, etc.

## Conclusions

Our study confirmed that a pro-inflammatory diet is associated with sarcopenia in Chinese community-dwelling older adults. Furthermore, we found that the highest DII score quartile was correlated with lower ASMI scores, gait speed, and test times on the 5-times sit-stand chair test. We also found that the association between the DII score and sarcopenia was mainly due to low intakes of dietary energy, protein, and anti-inflammatory foods, and not due to the high intake of pro-inflammatory foods. Inadequate nutrient intake may therefore be considered an urgent nutritional problem to address in the Chinese elderly population.

## Supplementary Information

**Additional file 1: Table 1S.** Diagnosis of sarcopenia using EWGSOP2. **Table 2S.** Multivariable-adjusted odds ratios (95% CIs) for sarcopenia defined by EWGSOP2 and its diagnostic parameters across DII quartile categories.

## Data Availability

Data will be available upon request from the corresponding author.
