# Supplementary material for: The association of dietary inflammatory potential with sarcopenia in Chinese community-dwelling older adults
Source: BMC Geriatr. 2023 May 10;23:281. doi: 10.1186/s12877-023-03938-7 (PMC10173667; doi:10.1186/s12877-023-03938-7)
Supplement: Supplementary file 1 — Additional file 1: Table 1S. Diagnosis of sarcopenia using EWGSOP2. Table 2S. Multivariable-adjusted odds ratios (95% CIs) for sarcopenia defined by EWGSOP2 and its diagnostic parameters across DII quartile categories. [file 12877_2023_3938_MOESM1_ESM.docx]

**Table 1S.** Diagnosis of sarcopenia using EWGSOP2

|  |  | N(%) |
| --- | --- | --- |
| Sarcopenia | | 30 (5.82) |
| Low handgrip strength | | 68 (13.20) |
| Low 5-times sit-stand chair test | | 69 (13.40) |
| Low gait speed | | 90 (17.48) |
| Low SPPB | | 19 (3.69) |
| Low ASMI | | 76 (14.76) |

**Table 2S.** Multivariable-adjusted odds ratios (95% CIs) for sarcopenia defined by EWGSOP2 and its diagnostic parameters across DII quartile categories.

| Model | DII, OR (95%CI) | | | | **p-trend** |
| --- | --- | --- | --- | --- | --- |
|  | Quartile 1  (n = 128) | Quartile 2  (n = 129) | Quartile 3  (n = 129) | Quartile 4  (n = 129) |  |
| Sarcopenia | | | | |  |
| Model 1 | 1 | 1.512 (0.416, 5.491) | 2.050 (0.601, 6.985) | 3.179 (0.997, 10.137) | 0.032 |
| Model 2 | 1 | 2.066 (0.475, 8.982) | 3.046 (0.747, 12.430) | 5.129 (1.328, 19.814) ^*^ | 0.009 |
| Model 3 | 1 | 2.273 (0.486, 10.619) | 4.210 (0.934, 19.973) | 9.025 (1.941, 41.965) ^*^ | 0.002 |
| Low muscle mass (ASMI) | | | | |  |
| Model 1 | 1 | 1.059 (0.519, 2.160) | 1.128 (0.557, 2.284) | 1.342 (0.676, 2.667) | 0.386 |
| Model 2 | 1 | 1.173 (0.504, 2.727) | 1.482 (0.633, 3.422) | 1.979 (0.865, 4.527) | 0.084 |
| Model 3 | 1 | 1.260 (0.522, 3.037) | 1.608 (0.642, 4.030) | 2.033 (0.782, 5.287) | 0.126 |
| Low muscle strength (handgrip strength) | | | | |  |
| Model 1 | 1 | 0.744 (0.353, 1.568) | 0.685 (0.320, 1.464) | 1.326 (0.677, 2.597) | 0.419 |
| Model 2 | 1 | 0.802 (0.372, 1.730) | 0.781 (0.358, 1.703) | 1.522 (0.759, 3.053) | 0.229 |
| Model 3 | 1 | 0.773 (0.348, 1.717) | 0.793 (0.350, 1.789) | 1.489 (0.680, 3.258) | 0.304 |
| Low muscle performance (SPPB) | | | | |  |
| Model 1 | 1 | 0.992 (0.243, 4.055) | 0.992 (0.243, 4.055) | 1.779 (0.508, 6.231) | 0.349 |
| Model 2 | 1 | 0.936 (0.224, 3.908) | 0.818 (0.194, 3.451) | 1.658 (0.463, 5.946) | 0.431 |
| Model 3 | 1 | 0.675 (0.137, 3.327) | 0.925 (0.192, 4.448) | 2.597 (0.550, 10.462) | 0.212 |
| Low muscle performance (6 m walk test) | | | | |  |
| Model 1 | 1 | 1.811 (0.905, 3.622) | 1.635 (0.810, 3.300) | 2.185 (1.108, 3.958) ^*^ | 0.078 |
| Model 2 | 1 | 1.769 (0.866, 3.612) | 1.520 (0.730, 3.148) | 1.960 (0.963, 3.988) | 0.106 |
| Model 3 | 1 | 1.846 (0.887, 3.844) | 1.694 (0.787, 3.647) | 2.105 (0.964, 4.593) | 0.092 |
| Low muscle performance (5-times sit-stand chair test) | | | | |  |
| Model 1 | 1 | 1.400 (0.617, 3.177) | 1.952 (0.894, 4.260) | 2.308 (1.074, 4.960) ^*^ | 0.020 |
| Model 2  Model 3 | 1 | 1.373 (0.597, 3.157) | 1.705 (0.766, 3.796) | 2.257 (1.038, 4.911) ^*^ | 0.030 |
|  | 1 | 1.490 (0.631, 3.519) | 2.019 (0.863, 4.724) | 2.686 (1.128, 6.393) ^*^ | 0.020 |
